# Intertwined nematic and *d*-wave superconductive orders in optimally doped La_1.84_Sr_0.16_CuO_4_ thin films

**DOI:** 10.1093/nsr/nwag309

**Published:** 2026-05-27

**Authors:** Gangfan Chen, Yichi Zhang, Guangyu Xi, JingYi Shen, Jie Wu

**Affiliations:** Department of Physics, School of Science, Westlake University, Hangzhou 310024, China; Research Center for Industries of the Future, Westlake University, Hangzhou 310024, China; Key Laboratory for Quantum Materials of Zhejiang Province, School of Science, Westlake University, Hangzhou 310024, China; School of Physics, Zhejiang University, Hangzhou 310027, China; Department of Physics, School of Science, Westlake University, Hangzhou 310024, China; Research Center for Industries of the Future, Westlake University, Hangzhou 310024, China; Key Laboratory for Quantum Materials of Zhejiang Province, School of Science, Westlake University, Hangzhou 310024, China; School of Physics, Zhejiang University, Hangzhou 310027, China; Department of Physics, School of Science, Westlake University, Hangzhou 310024, China; Research Center for Industries of the Future, Westlake University, Hangzhou 310024, China; Key Laboratory for Quantum Materials of Zhejiang Province, School of Science, Westlake University, Hangzhou 310024, China; School of Physics, Zhejiang University, Hangzhou 310027, China; Department of Physics, School of Science, Westlake University, Hangzhou 310024, China; Research Center for Industries of the Future, Westlake University, Hangzhou 310024, China; Key Laboratory for Quantum Materials of Zhejiang Province, School of Science, Westlake University, Hangzhou 310024, China; School of Physics, Zhejiang University, Hangzhou 310027, China; Department of Physics, School of Science, Westlake University, Hangzhou 310024, China; Research Center for Industries of the Future, Westlake University, Hangzhou 310024, China; Key Laboratory for Quantum Materials of Zhejiang Province, School of Science, Westlake University, Hangzhou 310024, China

**Keywords:** cuprate superconductivity, electronic nematicity, superconducting fluctuation, *d*-wave superconductive order

## Abstract

The anisotropy of the superconducting state and superconducting fluctuations in the CuO_2_ plane is directly related to the superconducting mechanism of copper oxide superconductors and is therefore pivotal for understanding high-temperature superconductivity. Here, we integrated the high-precision angle-resolved resistivity measurement with a rotatable in-plane magnetic field to systematically study the angular dependence of superconducting fluctuations in optimally doped La_1.84_Sr_0.16_CuO_4_ (LSCO) thin films. By independently controlling the directions of the current and the magnetic field, we are able to isolate the magneto-resistivity contributed by the superconducting vortex motion and distinguish excitations from nematic superconductivity and *d*-wave superconductive order based on their respective *C*_2_ and *C*_4_ symmetries. Signatures of two intertwined superconductive orders are also evident in the measured angular dependence of the critical current. A *T*–*B* phase diagram of different types of superconducting fluctuations is determined. These findings are closely related to other intriguing phenomena, such as pair density wave, charge density wave, and local pairing.

## Teaser

By using the angle-resolved resistivity method, a study reveals intertwined nematic and d-wave superconductive orders in optimally doped La1.84Sr0.16CuO4 thin films.

## INTRODUCTION

The lattice structure of copper oxide superconductors consists of single or multiple CuO_2_ planes sandwiched between charge reservoir layers, with high-temperature superconductivity residing at the CuO_2_ planes [1–3]. CuO_2_ planes in different layers are coupled via Josephson coupling, and the out-of-plane and in-plane directions exhibit significant anisotropy, making the superconductivity quasi-two-dimensional. Thus, the symmetry of the superconducting and normal states in the CuO₂ plane is crucial for understanding the superconducting mechanism. Unlike the normal state of conventional superconductors, which behaves like a Fermi liquid and is spatially isotropic, the normal state of copper oxide superconductors has been shown to spontaneously break the rotational symmetry and is anisotropic in the CuO_2_ plane with *C*_2_ symmetry [4–10]. The nature of this electronic nematic normal state and its role in unconventional superconductivity remain fundamental open questions in the field.

Signatures of uniaxial orders, such as charge/spin stripe order [11–19] and pair density wave (PDW) [20–23], have been detected in copper oxide superconductors by various techniques, including neutron scattering [11,12], scanning tunneling microscopy [18,19,21–23], optical pump-probe technique [15,16], muon spin rotation spectroscopy [17], etc. In principle, intertwinement of the PDW and nematic order with the *d*-wave superconductive order [24,25] allows the superconducting order parameter to take on various forms [26,27], for example, a nematic PDW state was identified in Bi_2_Sr_2_CaCu_2_O_8+x_ by Josephson tunneling microscopy [23]. To determine the actual order parameter and assess its universality across different families of copper oxide superconductors, it is imperative to elucidate the anisotropy of superconductivity within the CuO_2_ plane in real space.

Magneto-transport has been utilized to characterize nematic superconductivity in Fe-based [28,29] and nickelate superconductors [30]. The angular dependence of magneto-resistivity on the direction of the in-plane magnetic field reveals the anisotropy in the critical magnetic field and the spontaneous breaking of rotational symmetry in the superconducting state. These electrical transport experiments were carried out using the Corbino-shape configuration [29–31], so the measured magneto-resistivity was averaged over all the in-plane current directions. While this method is effective in retrieving the anisotropy induced by an external in-plane magnetic field, it overlooks the intrinsic anisotropy in resistivity corresponding to different current directions, for example, correlated angular oscillations in longitudinal and transverse resistivity with continuously varying current direction due to electronic nematicity [10,32].

To overcome this difficulty, we conceived and implemented the angle-resolved resistivity (ARR) method [32], which allows independent control of the in-plane directions of both excitation current and magnetic field. This enables us to distinguish the contributions to magneto-resistivity from various origins, such as electronic nematicity, the excitation of superconducting vortices, and superconducting fluctuations. The anisotropies of each effect were separated, and their correlations were mapped onto a *T*–*B* phase diagram. The observed *C*_2_ and *C*_4_ symmetry in superconducting fluctuation and critical current density suggest the coexistence of both nematic and *d*-wave superconductive order parameters.

## RESULTS

### Film synthesis and the ARR methodology

**Figure 1.**
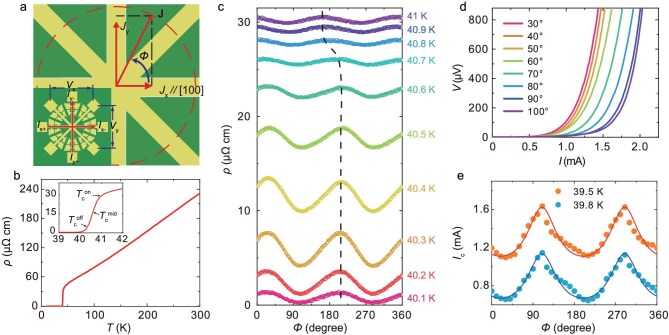
Anisotropic electrical transport of LSCO in the absence of magnetic field. (a) The schematic drawing of the pattern used for the ARR measurements. A complete view of the entire pattern is shown in the lower-left corner, laid on top of a magnified view of the central region. The *x* and *y* components of the current density **J**, *J_x_* and *J_y_*, are independently controlled by the currents injected into the paired contacts *I*_*x*+_ and *I*_*x*−_, and the pair *I*_*y*+_ and *I*_*y*−_. By letting ${I}_{x}\ = \ {I}_0\cos \phi$ and ${I}_{{y}}\ = \ {I}_0\sin \phi$, **J** at the cross section of the pattern can be rotated continuously in-plane, while the *x*- and *y*-components of the voltages, *V_x_* and *V_y_*, are measured simultaneously. (b) The temperature-dependent longitudinal resistivity $\rho ( T )$, measured along the LSCO [100] direction, manifests sharp superconducting transition. The inset magnifies the superconducting transition with $T_{\mathrm{c}}^{{\mathrm{on}}}$ at 41.1 K, $T_{\mathrm{c}}^{{\mathrm{mid}}}$ at 40.6 K, and $T_{\mathrm{c}}^{{\mathrm{off}}}$ at 40.4 K. Here, $T_{\mathrm{c}}^{{\mathrm{on}}}$, $T_{\mathrm{c}}^{{\mathrm{mid}}}$, and $T_{\mathrm{c}}^{{\mathrm{off}}}$ denote the temperatures with 90%, 50%, and 10% of the normal state resistivity. (c) The angle-dependent $\rho ( {\phi} )$ at variable *T* around the superconducting transition evidence the electronic nematicity. Here, $\phi$ is the angle between **J** and the LSCO crystalline [100] direction. The dashed line traces the peaks in $\rho ( {\phi} ),$ illustrating the transition of nematic director due to nematic superconducting fluctuations. (d) The *I*–*V* curves at a fixed temperature (*T* = 39.5 K) taken at systematically varying $\phi$ clearly show that the critical current *I*_c_ depends on the angle $\phi$. (e) The angle-dependent ${I}_{\rm c}( {\phi} )$ at two representative temperatures manifest that the superconductivity is strongly anisotropic in-plane and its *C*_2_ symmetry is correlated with the nematicity, that is, the peaks in ${I}_{\rm c}( {\phi} )$ corresponds to the valleys in$\ \rho ( {\phi} )$ at *T* = 40.1–40.7 K range. The solid circles are experimental data and the solid curves are the best fittings according to the expression ${I}_{\mathrm{c}}( {\phi} ) = a/( {b + {\mathrm{cos}}( {2\phi - \alpha } )} )$.

Optimally doped 26 nm LSCO (*x* = 0.16) films were synthesized on SrLaAlO_4_(001) (LSAO) substrates using the pulsed laser deposition technique. The high crystalline quality was confirmed by a clear *in situ* reflection high energy electron diffraction (RHEED) pattern, oscillations in RHEED spot intensity during deposition, and *ex situ* X-ray diffraction spectrum. A post-growth annealing of LSCO film was performed *in situ* at 450°C for 40 min to remove excess oxygen. The LSAO surface before deposition and the LSCO film surface after deposition were atomic flat, verified by atomic force microscopy. The films were patterned by standard UV-lithography to create the structure shown in Fig. 1a for electric transport measurements. An optical microscope image of a patterned LSCO film, together with further details on film synthesis, characterization, and fabrication are provided in the Methods section and the Supplemental Material.

To control the direction of current, we take advantage of the vector property of the current density **J**. By independently controlling the *x* and *y* components of **J**, *J_x_* and *J_y_*, we can continuously rotate **J** in the *x*–*y* plane at the cross section of the pattern (Fig. 1a) (here the *x*- and *y*-axes correspond to the LSCO [100] and [010] directions, respectively) while simultaneously measure the corresponding voltages, *V_x_* and *V_y_*. Then, the longitudinal and transverse resistivities with respect to **J**, $\rho $ and ${\rho }_{\mathrm{T}}$, are then obtained from the corresponding *V*_L_ and *V*_T_ by the relations $\rho = {V}_{\mathrm{L}}/I$ and ${\rho }_{\mathrm{T}} = {V}_{\mathrm{T}}/I$, where *I* is the applied current. Projecting *V*_L_ and *V*_T_ onto the *x*- and *y*-axis yields the relations ${V}_{\mathrm{L}} = {V}_{\mathrm{x}}{\mathrm{cos}}\phi + {V}_{\mathrm{y}}{\mathrm{sin}}\phi$ and ${V}_{\mathrm{T}} = {V}_{\mathrm{x}}{\mathrm{sin}}\phi - {V}_{\mathrm{y}}{\mathrm{cos}}\phi$, where $\phi$ is the angle between **J** and the *x*-axis. Varying $\phi$ from 0° to 360° by rotating **J** in-plane yields the complete angular dependence of $\rho ( {\phi} )$ and ${\rho }_{\mathrm{T}}( {\phi} )$. The angular resolution is only limited by the output electronic precision so it can be unprecedented high (<0.01°) if needed. Our sample holder is designed to be rotatable relative to the applied magnetic field, **B**, so that the direction of **B** can be varied continuously in-plane. In this way, the directions of **J** and **B** are controlled independently, enabling the anisotropic dependence of magneto-resistivity on each to be separated.

The effectiveness of the ARR methodology was verified in our previous publication [32]. There, a 10 nm thick gold control sample manifested faint angular oscillations (${\mathrm{\Delta }}R/\bar{R}$ < 0.05%) in the measured longitudinal and transverse resistance, $R( {\phi} )$ and ${R}_{\mathrm{T}}( {\phi} )$, which were attributed to inevitable small film thickness variations. This demonstrated that the ARR method is highly sensitive in detecting anisotropy in electric transport. Additionally, similar lithography pattern and measurement scheme have been used to study directional superconducting vortex motion [33], or intentionally introduced anisotropy [34].

### Electronic nematicity and nematic superconductivity in the absence of the magnetic field

A sharp superconducting transition is observed in the synthesized LSCO (*x* = 0.16) film, with $T_{\mathrm{c}}^{{\mathrm{on}}}$ at 41.1 K, $T_{\mathrm{c}}^{{\mathrm{mid}}}$ at 40.6 K, and $T_{\mathrm{c}}^{{\mathrm{off}}}$ at 40.4 K (Fig. 1b). Here, $T_{\mathrm{c}}^{{\mathrm{on}}}$, $T_{\mathrm{c}}^{{\mathrm{mid}}}$, and $T_{\mathrm{c}}^{{\mathrm{off}}}$ are defined as the onset, midpoint, and offset temperatures corresponding to 90%, 50%, and 10% of the normal state resistivity, respectively. The narrow transition width and high *T*_c_ value indicate the high quality and homogeneity of the film. Notably, $\rho ( {\phi} )$ slightly deviates from the linear-*T* dependence at low temperatures, which is due to the slight deviation from the optimal doping. In pulsed laser deposition, the chemical composition of the synthesized film may differ from that of the target because of differences in the evaporation temperatures of constituent elements. The resultant uncertainty in chemical doping is about 0.01, so the chemical doping of the LSCO film is actually 0.16 ± 0.01. Since the superconducting dome is flat near the optimal doping, *T*_c_ is not sensitive to small variations in doping. $\rho ( {\phi} )$, in contrast, shows noticeable deviation from linear-*T* dependence when the film is slightly under- or overdoped.

The angle-dependent $\rho ( {\phi} )$ at zero magnetic field shows clear angular oscillations as **J** varies its direction in the CuO_2_ plane (Fig. 1c). The 180° periodicity in $\phi$ evidences that the rotational symmetry is spontaneously broken and reduced to *C*_2_ symmetry. The $\rho ( {\phi} )$ and correlated ${\rho }_{\mathrm{T}}( {\phi} )$ oscillations originate from electronic nematicity and can be well fitted by the expressions $\rho ( {\phi} ) = \bar{\rho } + {\mathrm{\Delta }}\rho {\mathrm{cos}}[ {2( {\phi - \alpha } )} ]$ and ${\rho }_{\mathrm{T}}( {\phi} ) = {\mathrm{\Delta }}\rho {\mathrm{sin}}[ {2( {\phi - \alpha } )} ]$ (see the Supplemental Material for more details). Here, $\bar{\rho } = ( {{\rho }_a + {\rho }_b} )/2$ and ${\mathrm{\Delta }}\rho = ( {{\rho }_a - {\rho }_b} )/2$. ${\rho }_a$ and ${\rho }_b$ are the resistivities along two orthogonal symmetric axes associated with electronic nematicity, also referred to as the ‘nematic director’. Mathematically, ${\rho }_a$ and ${\rho }_b$ are the diagonal elements of the rank-2 resistivity tensor. $\alpha $ is the angle between the nematic director and the *x*-axis, as the nematicity is not necessarily aligned with the lattice. The deviation of these expressions and studies on electronic nematicity in the normal state of LSCO can be found in our previous publications [10,32] and the Supplemental Material. The focus of the current work, however, is on the transition from the superconducting to the normal state and the vestigial order in superconducting fluctuations. It should also be mentioned that contact misalignment and film inhomogeneity can give rise to a nonzero transverse resistivity. However, these extrinsic factors occur randomly from place to place, and their effects should be independent on *T* or *B*, which are against the observed systematic evolution of $\rho ( {\phi} )$ and ${I}_{\mathrm{c}}( {\phi} )$ with *T* and *B*. For a thorough discussion on contact misalignment and film inhomogeneity and how they are ruled out as the origin of the observed ${\rho }_{\mathrm{T}}( {\phi} )$, please refer to the Methods section of Ref. [10].

The peaks in $\rho ( {\phi} )$ shift abruptly as *T* lowers across $T_{\mathrm{c}}^{{\mathrm{on}}}$ (indicated by the dashed line in Fig. 1c). Concomitantly, the amplitude of $\rho ( {\phi} )$ oscillations is augmented significantly for *T* < $T_{\mathrm{c}}^{{\mathrm{on}}}$. Both facts imply that the underlying mechanisms responsible for electronic nematicity are different for the normal and superconducting fluctuating (SF) states [35]. The enhanced nematicity in the SF state is attributable to the vestigial nematicity in superconductivity.

The nematic nature of superconductivity is corroborated by the *I*–*V* curves taken below $T_{\mathrm{c}}^{{\mathrm{off}}}$. From the representative *I*–*V* curves for $30^\circ \ \le \ \phi\ \le \ 100^\circ $, it is evident that the critical current *I*_c_ varies appreciably with $\phi$ (Fig. 1d). The retrieved ${I}_{\mathrm{c}}( {\phi} )$ is strongly anisotropic, with the maximum *I*_c_ being roughly twice the minimum *I*_c_ (Fig. 1e). ${I}_{\mathrm{c}}( {\phi} )$ can be well fitted by the expression ${I}_{\mathrm{c}}( {\phi} ) = a/( {b + {\mathrm{cos}}( {2\phi - \alpha } )} )$, where *a, b* and $\alpha $ are the fitting parameters. Moreover, ${I}_{\mathrm{c}}( {\phi} )$ and $\rho ( {\phi} )$ are closely correlated: both have a 180° periodicity in $\phi$, and the peaks in ${I}_{\mathrm{c}}( {\phi} )$ ($\phi$ = 110° and 290°) correspond to the valleys in $\rho ( {\phi} )$. The lower resistivity and higher critical current imply that superconductivity is most robust along $\phi$ = 110°.

### Magneto-transport under an in-plane magnetic field

**Figure 2.**
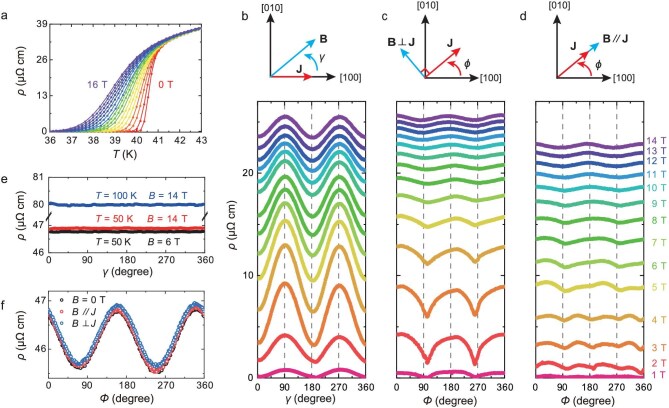
Anisotropic magneto-resistivity of LSCO. (a) The resistivity $\rho ( T )$ under incremental magnetic field along *x*-axis (LSCO [100] direction) from 0 to 16 T in 1 T step. (b) The longitudinal resistivity $\rho ( {\phi = {0}^{\mathrm{o}},\gamma } )$ under varying magnetic fields B from 1 to 14 T in 1 T step. The angle $\gamma $ between **B** and *x*-axis varies in-plane while the current flow **J** is fixed to be along the *x*-axis. The dominating ${\sin }^2\gamma $ oscillations originate from vortex motions, driven by the Lorentz force on superconducting vortices. (c) With **J** perpendicular to **B** (**J**⊥**B**), that is, $\gamma = \phi + \ {90}^{\circ}$, $\rho ( {\phi,\gamma = \phi + {{90}}^{\circ}} )$ are under the maximized influence of the Lorentz force. (d) With **J** in parallel to **B** (**J**//**B**), that is, $\gamma = \phi$, the Lorentz force diminishes. $\rho ( {\phi,\gamma = \phi} )$ manifest 4-fold symmetry at low magnetic field (1 T ≤ *B* ≤ 4 T), and 2-fold symmetry at higher magnetic field (5 T ≤ *B* ≤ 14 T). The temperature for panels b, c, and d is 40 K that is below $T_{\mathrm{c}}^{{\mathrm{off}}}$ at zero magnetic field. (e) As a comparison, $\rho ( {\phi = {0}^{\circ},\gamma } )$ shows no angular dependence on $\gamma $ once superconducting fluctuations are completely absent or suppressed, evidencing that the magneto-resistivity of the normal state is nearly independent of the magnetic-field orientation. (f) $\rho ( {\phi,B = 0} )$ at zero *B*-field has the same dependence on $\phi$ as $\rho ( {\phi,\gamma = \phi + {{90}}^{\circ}} )$ and $\rho ( {\phi,\gamma = \phi} )$ taken at *B* = 5 T for the **J**⊥**B** and **J**//**B** measurement schemes, respectively. The temperature, *T* = 50 K, is the same for all plots. This clearly shows that once the LSCO film is in the normal state, $\rho ( {\phi,B = 0} )$, $\rho ( {\phi,\gamma = \phi + {{90}}^{\circ}} )$ and $\rho ( {\phi,\gamma = \phi} )$ are all dictated by the electronic nematicity of the normal state and is insensitive to the direction of **B**.

Applying a magnetic field suppresses superconductivity and exposes the hidden states below $T_{\mathrm{c}}^{{\mathrm{off}}}$. In the presence of the magnetic field, the width of superconducting transition broadens and $T_{\mathrm{c}}^{{\mathrm{off}}}$ is lowered to 37.6 K at 16 T field (Fig. 2a), the maximum magnetic field accessible in our lab. The motion of superconducting vortex dissipates energy and is resistive to current flow. To distinguish this contribution from others, for example, quasiparticle excitation and superconducting phase fluctuation, we measured the angle-dependent magneto-resistivity $\rho ( {\phi,\gamma } )$ using three different schemes. Here, $\gamma $ is the angle between **B** and *x* axis, while **B** is always kept in-plane.

In the first scheme (Fig. 2b), **J** is fixed to be along *x* axis and $\gamma $ changes from 0° to 360°. The Lorentz force acted on superconducting vortices by the magnetic field is ${F}_{\mathrm{L}} = {\mathrm{\bf J}} \times {\mathrm{\bf B}}/c$, so the induced vortex motion v is normal to the film [36]. Consequently, this induces an electric field of magnitude ${\mathrm{\bf E}} = {\mathrm{\bf B}} \times {\mathrm{\bf v}}/c$ that is in-plane and transverse to **B**. Thus, the resultant longitudinal magneto-resistivity along **J** should have the relation $\rho ( {\phi = {0}^{\circ},\gamma } ) \propto {\mathrm{si}}{{\mathrm{n}}}^2\gamma $. This is consistent with the measured $\rho ( {\phi = {0}^{\circ},\gamma } )$ at variable *B* (Fig. 2b). $\rho ( {\phi = {0}^{\circ},\gamma } )$ is dominated by ${\mathrm{si}}{{\mathrm{n}}}^2\gamma $ oscillation, whose amplitude declines with *B*. In comparison, $\rho ( {\phi = {0}^{\circ},\gamma } )$ of the normal state, which is free of superconducting vortex, shows negligible angular oscillations (Fig. 2e). Thus, this confirms that the $\rho ( {\phi = {0}^{\circ},\gamma } )$ oscillations originate from vortex motion. More measurements and discussions on $\rho ( {\phi = {0}^{\circ},\gamma } )$ are provided in the Supplemental Material.

This scheme is widely used in in-plane angular magnetoresistance (AMR) measurement, where the current flow is fixed in direction in a Hall-bar geometry and the magnetic field is rotated in-plane relative to the current. A 2-fold in-plane AMR was reported in underdoped LSCO [37], YBa_2_Cu_3_O_6+x_ [13], and electron-doped La_2−x_Ce_x_CuO_4_ [38], while a 4-fold AMR was found in lightly electron-doped Pr_1.3−x_La_0.7_Ce_x_CuO_4_ [39], Nd_2−x_Ce_x_CuO_4_ [40], and Pr_2−x_Ce_x_CuO_4_ [41]. The 2-fold AMR is suggested to be associated with spin ordering, whereas the 4-fold AMR is attributed to a magnetic-field-induced spin flop transition. These findings, however, are fundamentally different from the ${\mathrm{si}}{{\mathrm{n}}}^2\gamma $ oscillations shown in Fig. 2b. The AMR results mentioned above pertain to underdoped cuprates, most of which are non-superconducting. In contrast, the LSCO film in our study is optimally doped. More importantly, the 2-fold or 4-fold AMR observed in previous studies occurs at temperatures much higher than *T*_c_ and diminishes with the static or quasistatic antiferromagnetic order [41]. Conversely, ${\mathrm{si}}{{\mathrm{n}}}^2\gamma $ oscillations in our LSCO film are substantial in amplitude only near *T*_c_ and disappear completely once superconducting fluctuation vanishes (Fig. 2e). Therefore, the ${\mathrm{si}}{{\mathrm{n}}}^2\gamma $ oscillations should be associated with superconducting vortices rather than antiferromagnetic order.

In order to probe the symmetry of superconducting order parameters, it is necessary to fix the contribution of mobile superconducting vortex to magneto-resistivity by fixing the relative angles between **J** and **B**. Hence, the second measurement scheme is to keep **B** perpendicular to **J** and rotate both of them simultaneously so that the Lorentz force due to the magnetic field remains maximized (Fig. 2c). The third scheme, on the other hand, is to keep **B** parallel to **J** during rotation to completely remove the influences of the Lorentz force (Fig. 2d). The measured $\rho ( {\phi,\gamma = \phi + {{90}}^{\circ}} )$ and $\rho ( {\phi,\gamma = \phi} )$ show remarkable differences at low magnetic fields but manifest similar ${\mathrm{sin}} ( {2\phi + {{114}}^{\circ}} )$ oscillations at high magnetic fields (Fig. 2c and d). It is worth noting that the peak angles ($\phi = {168}^{\circ}$ and ${348}^{\circ}$) in $\rho ( {\phi,\gamma = \phi + {{90}}^{\circ}} )$ differ from those in $\rho ( {\phi = {0}^{\circ},\gamma } )$ but identical to those in $\rho ( {\phi,\gamma = \phi} )$ at high magnetic fields, for example, *B* = 14 T. Moreover, they are also the same as the peak angles in $\rho ( {\phi} )$ at 41 K in the absence of a magnetic field. This is no coincidence. It illustrates that $\rho ( {\phi,\gamma = \phi + {{90}}^{\circ}} )$ and $\rho ( {\phi,\gamma = \phi} )$ at large **B**-fields are dominated by their dependences on **J** and, therefore, are governed by electronic nematicity in the normal state. Once superconducting fluctuations are suppressed by elevated temperatures, or magnetic fields, or both, $\rho ( {\phi,\gamma = \phi + {{90}}^{\circ}} )$ and $\rho ( {\phi,\gamma = \phi} )$ are dictated by the nematicity in the normal state, which is insusceptible to the in-plane magnetic field (Fig. 2f).

As the magnetic field decreases and superconducting fluctuations strengthen, two dips at $\phi$ = 102° and 260° gradually develop in $\rho ( {\phi,\gamma = \phi + {{90}}^{\circ}} )$ (Fig. 2c). This seems to contradict the expectation that the magneto-resistivity induced by vortex motion, being proportional to **J** × **B**, should be angle-independent in the second measurement scheme. It may be related to the drifting of vortices along the current flow, which could be anisotropic in-plane and influenced by randomly distributed pinning centers along the current’s path. Further investigations are required to elucidate the mechanism underlying this observation.

### Coexistence of *C*_2_ and *C*_4_ symmetries in the SF state

**Figure 3.**
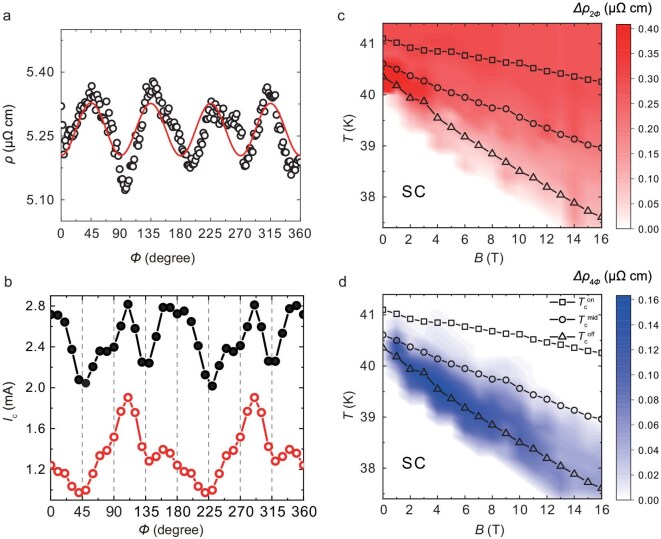
*B*–*T* phase diagram for the *C*_2_ and *C*_4_ anisotropies in superconducting fluctuations. (a) The angular oscillations in $\rho ( {\phi,\gamma = \phi} )$ taken at *T* = 38.2 K and *B* = 14 T with **J**//**B**. The experimental data (black solid circles) can be well fitted by the expression $\rho ( {\phi,\gamma = \phi} ) = \bar{\rho } + {\mathrm{\Delta }}\rho {\mathrm{cos}}[ {4( {\phi - \alpha } )} ]$ (red lines). (b) The critical current ${I}_c( {\phi,\gamma = \phi} )$ taken at *T* = 37 K (black open circles) and 37.5 K (red open circles) at *B* = 8 T. (c and d) The *B*–*T* phase diagram for the *C*_2_ and *C*_4_ components of the angle-dependent resistivity $\rho ( {\phi,\gamma = \phi} )$. The amplitudes ${\mathrm{\Delta }}{\rho }_{2\phi}$ and ${\mathrm{\Delta }}{\rho }_{4\phi}$ are retrieved from the expression $\rho ( {\phi,\gamma = \phi} ) = \bar{\rho } + {\mathrm{\Delta }}{\rho }_{2\phi}{\mathrm{cos}}[ {2( {\phi - \alpha } )} ] + {\mathrm{\Delta }}{\rho }_{4\phi}{\mathrm{cos}}[ {4( {\phi - \beta } )} ]$. The $T_{\mathrm{c}}^{{\mathrm{on}}}( B )$, $T_{\mathrm{c}}^{{\mathrm{mid}}}( B )$, and $T_{\mathrm{c}}^{{\mathrm{off}}}( B )$ curves denote the temperatures with 90%, 50%, and 10% of the normal state resistivity, respectively. ‘SC’ stands for the superconducting state.

In the absence of the Lorentz force and the resultant vortex motion, the measured $\rho ( {\phi,\gamma = \phi} )$ reflects the symmetry of superconducting order parameter. As *B* decreases, the symmetry of $\rho ( {\phi,\gamma = \phi} )$ changes from 2-fold to 4-fold (Fig. 2d), for example, at *B* = 3 T, the period of angular oscillation becomes 90°. Applying a stronger magnetic field shifts $T_{\mathrm{c}}^{{\mathrm{off}}}$ to lower temperature. Following the superconducting-to-normal phase boundary $T_{\mathrm{c}}^{{\mathrm{off}}}( B )$, the 4-fold oscillations are found to be ubiquitous in the vicinity of superconducting transition. For instance, the $\rho ( {\phi,\gamma = \phi} )$ taken at *T* = 38.2 K and *B* = 14 T (Fig. 3a) shows four peaks at $\phi$ = 45°, 135°, 225°, and 315° that correspond to the Cu–Cu bond directions of LSCO lattice.

In accord with $\rho ( {\phi,\gamma = \phi} )$ in the SF state, the critical current ${I}_{\mathrm{c}}( {\phi,\gamma = \phi} )$ for the superconducting state, with **B** parallel to **J**, manifests a mixture of 2-fold and 4-fold symmetries (Fig. 3b), in contrast to the 2-fold symmetric ${I}_{\mathrm{c}}( {\phi} )$ in the absence of magnetic field (Fig. 1e). The dips of ${I}_{\mathrm{c}}( {\phi,\gamma = \phi} )$ occur at $\phi$ = 45°, 135°, 225°, and 315° (Fig. 3b) that have one-to-one correspondence with the peaks in $\rho ( {\phi,\gamma = \phi} )$ (Fig. 3a). Thus, superconductivity is more robust along the Cu–O–Cu bond than the Cu–Cu bond direction, consistent with the widely accepted ${d}_{{x}^2 - {y}^2}$ superconducting gap symmetry for copper oxide superconductors.

In addition to the *C*_4_ component, it is evident that ${I}_{\mathrm{c}}( {\phi,\gamma = \phi} )$ also contains a *C*_2_ component, for example, two well-defined peaks in ${I}_{\mathrm{c}}( {\phi,\gamma = \phi} )$ emerge at $\phi$ = 110°, and 290° (Fig. 3b), which are the same angles corresponding to the valleys in $\rho ( {\phi} )$ at *T* = 40.1 K (Fig. 1c). Hence, the *C*_2_ component originates from the electronic nematicity in the SF state. The coexistence of *C*_4_ and *C*_2_ components implies that nematic superconductivity coexists with *d*-wave superconductivity in LSCO.

To map out an explicit phase diagram for states with *C*_2_ and *C*_4_ symmetries, we retrieved the amplitudes ${\mathrm{\Delta }}{\rho }_{2\phi}$ and ${\mathrm{\Delta }}{\rho }_{4\phi}$ from the best fits of the resistivity by the expression $\rho ( {\phi,\gamma = \phi} ) = \bar{\rho } + {\mathrm{\Delta }}{\rho }_{2\phi}{\mathrm{cos}}[ {2( {\phi - \alpha } )} ] + {\mathrm{\Delta }}{\rho }_{4\phi}{\mathrm{cos}}[ {4( {\phi - \beta } )} ]$ (Fig. 3c and d), where *α* and *β* are two fitting parameters standing for the phase offsets. In the *T*–*B* phase diagram, $T_{\mathrm{c}}^{{\mathrm{on}}}( B )$ and $T_{\mathrm{c}}^{{\mathrm{off}}}( B )$ denote the phase boundaries separating the superconducting, SF, and normal states. Remarkably, the phases with appreciable ${\mathrm{\Delta }}{\rho }_{4\phi}$ closely trace the $T_{\mathrm{c}}^{{\mathrm{off}}}( B )$ curve, implying that the *C*_4_ component emerges only in the vicinity of the superconducting state. The *C*_2_ component ${\mathrm{\Delta }}{\rho }_{2\phi}$, on the other hand, is weak where the *C*_4_ component is present but persists all the way to high *T* and *B*.

## DISCUSSION

The amplitude of 4-fold angular oscillation, ${\mathrm{\Delta }}{\rho }_{4\phi}$, is a measure of the amplitude of *d*-wave order parameter. In magneto-transport experiment, the 4-fold symmetry was observed in several superconducting copper oxides and was ascribed to the *d*-wave superconducting gap symmetry. Examples include the *c*-axis magneto-resistivity with variable in-plane magnetic field in Pb_2_Sr_2_Y_0.62_Ca_0.38_Cu_3_O_8_ [42] and La_1.6-*x*_Nd_0.4_SrCuO_4_ [43], in-plane torque anisotropy in Tl_2_Ba_2_CuO_6+δ_ [44], and angular dependence of the upper critical field in LSCO [45]. In other superconductors, the 4-fold symmetry manifested in magnetoresistance was also attributed to their superconducting symmetry, for example, in LiTi_2_O_4_ [31] and Nd_0.8_Sr_0.2_NiO_2_ [30]. Thus, it is no surprise that the 4-fold symmetry emerges in $\rho ( {\phi,\gamma = \phi} )$ of our optimally doped LSCO and ${\mathrm{\Delta }}{\rho }_{4\phi}$ serves as an indicator for the strength of the *d*-wave superconductive order.

The *d*-wave order parameter changes sign along two orthogonal anti-nodal directions. When global phase coherence is absent, the phase of the *d*-wave order parameter may vary spatially from place to place. Since the ARR method measures an averaged signal over a micrometer-scale region, it may be insensitive to such local *d*-wave pairing fluctuations. Only when the superconducting state becomes sufficiently well developed and the phase-coherence length becomes comparable to the dimension of the ARR device, does the signature of *d*-wave superconducting order become detectable in electrical transport measurements. This explains why the *C*_4_ component appears only below $T_{\mathrm{c}}^{{\mathrm{off}}}$.

The amplitude of 2-fold angular oscillation, ${\mathrm{\Delta }}{\rho }_{2\phi}$, is a measure of the order parameter of nematic superconductivity. Although a direct measurement of the nematicity in the superconducting state is not feasible via electric transport, superconducting fluctuation at the temperatures between $T_{\mathrm{c}}^{{\mathrm{on}}}( B )$ and $T_{\mathrm{c}}^{{\mathrm{off}}}( B )$ is clearly nematic that is an imminent consequence of nematic superconductivity. The nematicity may be related to the stripe phase or checkerboard order [11–19] and can couple to PDW order to form nematic PDW, as indicated by the recent Josephson tunneling microscopy [23].

**Figure 4.**
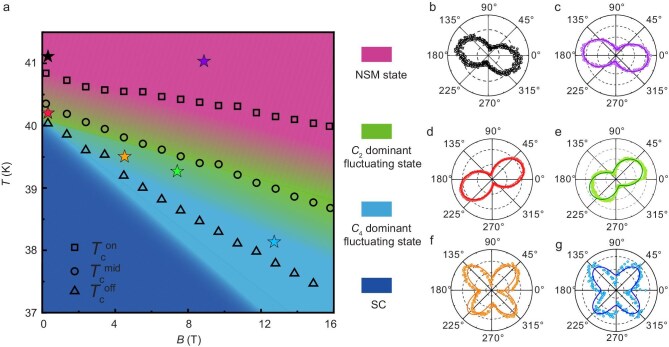
*B*–*T* phase diagram for superconducting fluctuations. (a) ‘SC’ stands for the superconducting state, ‘*C*_4_ dominant fluctuating state’ for the intermediate state with dominant *d*-wave superconductive order fluctuations, ‘*C*_2_ dominant fluctuating state’ for the intermediate state with dominant nematic superconducting fluctuations, and ‘NSM state’ for the nematic strange metal state. (b–g) The polar plots of $\rho ( {\phi,\gamma = \phi} )$ show the directors of *C*_2_ and *C*_4_ anisotropies at representative (*T, B*) values, with their corresponding locations marked by stars in panel a. The colors of the stars correspond one-to-one with the colors of the respective polar plots.

The phase diagram of the superconductive orders is summarized in Fig. 4a. As *T* or *B* increases, the superconducting state evolves into the intermediate state with dominant *d*-wave superconductive order fluctuations (‘*C*_4_ dominant fluctuating state’), then the intermediate state with dominant nematic superconducting fluctuations (‘*C*_2_ dominant fluctuating state’), and eventually the nematic strange metal state (‘NSM state’). The polar plots of $\rho ( {\phi,\gamma = \phi} )$ for these three states differ drastically. The ‘cloverleaf’ shape of $\rho ( {\phi,\gamma = \phi} )$ for the *C*_4_ dominant fluctuating state is aligned with the crystalline lattice, with $\rho ( {\phi,\gamma = \phi} )$ peaking along the Cu–Cu bond direction (Fig. 4f and g). Conversely, the directors of the ‘dumbbell’ shape of $\rho ( {\phi,\gamma = \phi} )$ for the *C*_2_ dominant fluctuating state (Fig. 4d and e) and the NSM state (Fig. 4b and c) are not aligned with the lattice. Notably, there is an abrupt rotation of the nematic director across the boundary between the *C*_2_ dominant fluctuating state and the NSM state.

We have also begun experiments on underdoped and overdoped LSCO films. The preliminary data shows that both *C*_2_ and *C*_4_ angular oscillations are present in both underdoped and overdoped LSCO in the **B**//**J** configuration, consistent with the behavior observed in the optimally doped LSCO. Thus, the main conclusion—the intertwinement of nematic and *d*-wave superconductive orders—is universal for superconducting states inside the superconducting dome. Similar intertwined orders have also been observed in bulk cuprates, for example, by scanning tunneling microscopy [23], suggesting that they are intrinsic to cuprates irrespective of whether they are in bulk or thin-film form. This is consistent with the quasi-two-dimensional nature of cuprates, which exhibit strong anisotropy between the CuO_2_ planes and the out-of-plane direction, while superconductivity is hosted in the CuO_2_ planes.

From these results, we conclude that nematic and *d*-wave superconductive orders coexist in high temperature superconducting copper oxides. The intertwining of electronic nematicity and superconductivity enhances the amplitude of nematicity and alters its director, as evidenced by the stronger nematicity in the *C*_2_ dominant state compared to the NSM state. This finding provides new insights into the pairing mechanism and the formation of phase coherence in high-temperature superconductivity.

## METHODS

### Film synthesis and device fabrication

The La_1.84_Sr_0.16_CuO_4_(001) thin films were epitaxially grown on SrLaAlO_4_(001) substrates by pulsed laser deposition using a 248 nm KrF laser. Prior to growth, the SrLaAlO_4_ substrate was annealed at 720°C for 20 min under an oxygen pressure of 10^−2^ Torr. During deposition, the substrate temperature was kept at 700°C with the oxygen pressure of 9 × 10^−2^ Torr. A laser fluence of 1 J/cm^2^ was used to ablate the target at a repetition frequency of 2 Hz. For post-annealing, the LSCO films were held at 450°C for 40 min in an oxygen pressure of 3.8×10^2^ Torr.

### Devices fabrication and characterization

The films were patterned by standard UV-lithography and ion milling to form the device shown in Fig. 1a. Then the electrodes (Au, 100 nm) were deposited via magnetron sputtering. X-ray diffraction experiments were conducted at room temperature using a Bruker D8-Advanced X-ray diffractometer with Cu ${K}_\alpha $ radiation ($\lambda $ = 1.5406 Å).

### Transport measurements

The electrical transport measurements were performed using a 16 T Physical Property Measurement System (PPMS-16T, Quantum Design) equipped with a rotator featuring an angular accuracy of 0.05°. For transport measurements, DC currents were supplied by Keithley 2450 sourcemeters, and DC voltages were measured using Keithley 2182A nanovoltmeters. The measuring current $I = 100{\mathrm{\ \mu A}}$.

## Supplementary Material

nwag309_Supplemental_File
