# Supplementary material for: Intertwined nematic and d-wave superconductive orders in optimally doped La1.84Sr0.16CuO4 thin films
Source: Natl Sci Rev. 2026 May 27;13(15):nwag309. doi: 10.1093/nsr/nwag309 (PMC13455619; doi:10.1093/nsr/nwag309)
Supplement: Supplementary file 1 [file nwag309_supplemental_file.docx]

**Intertwined nematic and *d*-wave superconductive orders in optimally-doped La_1.84_Sr_0.16_CuO_4_**

G.F. Chen^1, 2, 3, 4^, Y.C. Zhang^1, 2, 3, 4^, G.Y. Xi^1, 2, 3, 4^, J.Y. Shen^1, 2, 3, 4^, J. Wu^1, 2, 3*^

*^1^Department of Physics, School of Science, Westlake University, Hangzhou 310024, China*

*^2^Research Center for Industries of the Future, Westlake University, Hangzhou 310024, China*

*^3^Key Laboratory for Quantum Materials of Zhejiang Province, School of Science, Westlake University, Hangzhou, 310024, China*

*^4^School of Physics, Zhejiang University, Hangzhou 310027, China*

*Author to whom correspondence should be addressed:* [*wujie@westlake.edu.cn*](mailto:wujie@westlake.edu.cn)

**Supplementary information**

**1. Structural and morphological characterizations**

Structural and morphological characterizations confirm the high quality of synthesized single-crystalline La_1.84_Sr_0.16_CuO_4_ (LSCO) films.

The crystalline structure of the LSCO film is monitored *in situ* in real time using the reflection high energy electron diffraction (RHEED) apparatus during film deposition. The streak-like RHEED pattern and the oscillations in RHEED spot intensity over time (Fig. S1) confirm that the film grows in the desired layer-by-layer mode and that the LSCO film is single-crystalline with a flat surface throughout the growth process. It is remarkable that the RHEED spot intensity does not decay even after 20 cycles of oscillations—a fingerprint of ideal film synthesis.

**Figure S1 │ The oscillations in RHEED spot intensity over deposition time.** The intensity does not decay even after 20 cycles of oscillations. The inset shows the RHEED pattern during growth.

The lattice structure of the LSCO film is further examined *out situ* using X-ray diffraction (XRD). The pronounced XRD peak corresponding to LSCO (006) diffraction peak is manifested in the $2\theta$-$\omega$ scan (Fig. S2), confirming the single-crystallinity of the LSCO film. The measured *c*-axis lattice constant is 1.326 nm. The periodic fringe peaks reflect that both the LSCO/LaSrAlO4 interface and the LSCO surface are sharp enough to produce the interference of X-rays. The film thickness, determined from the periodicity of the fringe peaks, is 26 nm that agrees nicely with the thickness measured by RHEED oscillations.

**Figure S2 │ The XRD** $\boldsymbol{2}\boldsymbol{\theta}$**-**$\boldsymbol{\omega}$ **scan of the LSCO film.**

The morphology of the LSCO film is studied using atomic force microscopy (AFM). The AFM image of a 20 uc (~26 nm) thick LSCO film shows clear atomic steps (Fig. S3). The overall root-mean-square (r.m.s.) surface roughness is about 0.377 nm, which should be contrasted with the *c*-axis lattice constant, 1.326 nm. Thus, the surface roughness is much less than the height of a single unit cell, implying that the LSCO film is atomically flat.

**Figure S3 │ AFM image taken from a 26 nm thick LSCO film.** Atomic steps are clearly visible. The rms roughness is about 0.377 nm.

**2. Homogeneity of superconductivity**

The sharpness of superconducting transition is a sensitive measure of the homogeneity of superconductivity. We facilitate the two-coil mutual inductance technique^45-47^ to measure the diamagnetic signal due to Meissner effect.

The voltage across the pick-up coil, *V*_p_, have both in-phase and off-phase components, Re*V*_p_ and Im*V*_p_, in response to the *ac* currents in the drive coil, which generates an *ac* magnetic field penetrating through the pick-up coil. Re*V*_p_ drops abruptly as soon as the LSCO film enters the superconducting state, and the screening supercurrent is excited by the *ac* magnetic field (Fig. S4). Simultaneously, Im*V*_p_ manifests a pronounced peak right below *T*_c_, due to energy dissipation consumed by superconducting fluctuations. The full width at half maximum (FWHM) of the Im*V*_p_(*T*) peak is around 1 K, which is a good estimate of *T*_c_ spread in our 5 mm × 5mm LSCO film.

**Figure S4 │ Homogeneity of superconductivity revealed by the mutual inductance measurement on a 26 nm thick LSCO film.** *V*_p_ is the voltage across the pick-up coil, which is proportional to the mutual inductance. The real and imaginary components of *V*_p_, Re*V*_p_ and Im*V*_p_, correspond to the blue and red curves line respectively. The FWHM of the Im*V*_p_(*T*) peak, *ΔT*, is 1 K and the superconducting temperature, *T*_c_, is 38.3 K.

The film was patterned into the device (Fig. S5a) for angle-resolved resistivity (ARR) measurements in the central area (location A) and four-point measurements on hall bars (location B). The temperature dependent of the normalized resistance *R*(*T*)/*R*(300 K) of a LSCO film was compared before and after patterning (Fig. S5b). For the unpatterned film, resistance was measured using the van der Pauw method. After patterning, resistance was measured at two locations: one at the center of the pattern, and the other at one of the Hall bars. The normalized resistance, *R*(*T*)/*R*(300 K), overlaps with each other, with both exhibiting a superconducting transition at *T*_c_ ≈ 40 K. This confirms that the transport properties of the LSCO film—particularly its superconductivity—are not affected by the lithography process.

**Figure S5 │ The normalized resistance, *R*(*T*)/*R*(300 K), for a LSCO film before and after patterning. a,** A photo of a patterned LSCO film taken by optical microscopy. **b,** For the unpatterned film, resistance was measured using the van der Pauw method. After the patterning process, resistance was measured at two locations on the LSCO film, with their locations indicated in panel a. The three *R*(*T*)/*R*(300 K) curves overlap with each other, confirming that the influence of lithography on the film’s transport properties is negligible.

**3. Electronic nematicity of the normal state**

The anisotropic electrical transport due to electronic nematicity in the normal state of LSCO was elucidated in our previous publications^10, 32^. This is not the main focus of the current work, but, for the completeness of the work, here we present a brief summary together with the measurement results on the LSCO film, from which the data in Figs. 1-3 were collected.

The in-plane resistivity is a rank-2 tensor:

$\rho=\left( \begin{matrix} \rho_{a} & 0 \\ 0 & \rho_{b} \end{matrix} \right)$,

where $\rho_{a}$ and $\rho_{b}$ are resistivities along two principal axes $\boldsymbol{e}_{a}$ and $\boldsymbol{e}_{b}$, respectively. If $\rho_{a}=\rho_{b}$, then $\rho$ is simply a scalar multiplied by a unit matrix. In the presence of electronic nematicity, the in-plane rotational symmetry is spontaneously reduced to **C_2_** symmetry. Consequently, $\rho_{a}\neq\rho_{b}$. $\boldsymbol{e}_{a}$ and $\boldsymbol{e}_{b}$ are orthogonal to each other, and they are the symmetric axes of **C_2_** symmetry.

For currents applied in the direction making an angle $\phi$ from the $\boldsymbol{e}_{a}$axis, the resistivity tensor becomes

$$\rho^{'}=\hat{C}_{\phi}\rho\hat{C}_{\phi}^{-1}=\left( \begin{matrix} cos\phi& -sin\phi\\ sin\phi& cos\phi\end{matrix} \right)\left( \begin{matrix} \rho_{a} & 0 \\ 0 & \rho_{b} \end{matrix} \right)\left( \begin{matrix} cos\phi& sin\phi\\ -sin\phi& cos\phi\end{matrix} \right)$$

$=\left( \begin{matrix} \bar{\rho}+\Delta\rho\cos(2\phi) & \Delta\rho\sin(2\phi) \\ \Delta\rho\sin(2\phi) & \bar{\rho}-\Delta\rho\cos(2\phi) \end{matrix} \right)$.

Here $\bar{\rho}=(\rho_{a}+\rho_{b})/2$ and $\Delta\rho=(\rho_{a}-\rho_{b})/2$.

Then the longitudinal and transverse resistivity are given by

$\rho\left( \phi\right)=\bar{\rho}+\Delta\rho\cos(2\phi)$,

$\rho_{T}\left( \phi\right)=\Delta\rho\sin(2\phi)$.

Taking into consideration that the principal axes $\boldsymbol{e}_{a}$ and $\boldsymbol{e}_{b}$, in general, are not aligned with the crystallographic orientations [100] and [010], we denote the angle between $\boldsymbol{e}_{a}$ and [100] as $\alpha$. Then the expressions of $\rho(\phi)$ and $\rho_{T}(\phi)$ become:

$\rho\left( \phi\right)=\bar{\rho}+\Delta\rho\cos\left[ 2\left( \phi-\alpha\right) \right]$ … (1)

$\rho_{T}\left( \phi\right)=\Delta\rho\sin\left[ 2\left( \phi-\alpha\right) \right]$ … (2)

These expressions^10, 32^ agree nicely with experimental results.

For the LSCO film, from which the data in Figs. 1-3 were collected, $\rho(\phi)$ and $\rho_{T}(\phi)$ were measured for its normal state. The angular oscillations in $\rho(\phi)$ and $\rho_{T}(\phi)$ at *T* = 300 K can be simultaneously fitted by Expressions (1) and (2) using the same set of fitting parameters $\Delta\rho$ and $\alpha$ (Fig. S6a). The nematicity magnitude can be represented by $N\equiv\Delta\rho/\bar{\rho}$. And *N*(*T*) increases as *T* decreases (Fig. S6b), consistent with the expectation that the nematicity will strengthen as the thermal fluctuations get weaker at lower temperatures. The electronic nematicity behaves very differently in the presence of strong superconducting fluctuations as *T* approaches *T_c_*. $\rho_{T}(\phi)$, plotted in the polar coordinate system, intuitively show the “cloverleaf” shape for both *T* = 300 K and *T* = *T_c_* (Fig. S6c). Moreover, there is a relative rotation between two cloverleaf patterns, and the angular difference, $\Delta\alpha=\left| \alpha\left( T=300K \right)-\alpha\left( T=T_{c} \right) \right|$, is about 60°. This is consistent with our previous findings^10, 32^, implying that superconducting fluctuations are strongly nematic—a direct evidence for nematic superconductivity in LSCO.

**Figure S6 │ Electronic nematicity in LSCO film by ARR measurements. a**, $\rho(\phi)$ and $\rho_{T}(\phi)$ manifest angular oscillations with 180^o^ period—a reflection of the **C_2_** symmetry of electronic nematicity. The open dots are experimental data and the red lines are the best fittings using Expressions (1) and (2). **b**, The temperature dependence of nematicity magnitude, $N\equiv\Delta\rho/\bar{\rho}$. **c**, $\rho_{T}(\phi)$ plotted in the polar coordinate system at *T* = 300 K and *T* = *T*_c_. The blue color stands for positive value and the red, negative.

**4. Magneto-resistance contributed by superconducting vortex motion**

For type-II superconductors, such as LSCO, superconducting vortices form when the superconductor is within a magnetic field, which is above the critical magnetic field *H_c1_*. Each vortex is subject to the Lorentz force^35^,

$\boldsymbol{F}_{L}=\boldsymbol{\phi}_{0}\boldsymbol{\times J}$,

where $\boldsymbol{J}$ is the current density and $\boldsymbol{\phi}_{0}$ is the flux quantum.

For our first measurement scheme, both $\boldsymbol{B}$ and $\boldsymbol{J}$ are in-plane, and the angle between them is $\gamma$. Thus, the Lorentz force is out-of-plane and proportional to $sin\gamma$.

The induced vortex motion with the velocity, $\boldsymbol{v}$, is normal to the plane, and it cuts through the magnetic field, dissipating energy and generating an in-plane electric field,

$\boldsymbol{E}=\boldsymbol{v}\times\boldsymbol{B}/c$.

Therefore, the electric field longitudinal and transverse to $\boldsymbol{J}$, $E$ and $E_{T}$, have such dependencies on $\gamma$ as

$E\propto sin\gamma\cdot\sin\gamma\propto-\cos2\gamma$,

$E_{T}\propto sin\gamma\cdot\cos\gamma\propto\sin2\gamma$.

In accord, the longitudinal and transverse resistivities, $\rho(\phi=0^{o},\gamma)$ and $\rho_{T}(\phi=0^{o},\gamma)$, satisfy the relations:

$\rho(\phi=0^{o},\gamma)=\bar{\rho}-\Delta\rho\cos\left( 2\gamma\right)$ … (3)

$\rho_{T}\left( \phi=0^{o},\gamma\right)=\bar{\rho}_{T}+\Delta\rho\sin\left( 2\gamma\right)$ … (4)

Here $\bar{\rho}$ and $\bar{\rho}_{T}$ are the average longitudinal and transverse resistivities, respectively, and $\Delta\rho$ is a fitting parameter corresponding to the amplitude of angular oscillations. $\bar{\rho}_{T}$ is non-zero due to the presence of electronic nematicity at zero magnetic field.

Note that although Expressions (3) and (4) look similar to Expressions (1) and (2), the physics origins are drastically different: the former is due to the Lorentz force acted by the magnetic field; the latter is due to electronic nematicity of LSCO in the absence of the magnetic field.

The typical $\rho(\phi=0^{o},\gamma)$ and $\rho_{T}(\phi=0^{o},\gamma)$ in the superconducting fluctuating state can be well fitted by Expressions (3) and (4) (Fig. S7a). This confirms firmly that the observed angular oscillations using the first measurement scheme are contributed by superconducting vortex motions.

Furthermore, $\Delta\rho\left( B \right)$ at various temperatures are shown in Fig. S7b. For the superconducting state, e.g., *T* = 38 K, both the population of superconducting vortex and the induced *E* field are proportional to *B*. Consequently, $\Delta\rho$ increases with *B*. For the superconducting fluctuating state, e.g., *T* = 40.5 K, the residual superconductivity is severely suppressed by *B* so that $\Delta\rho$ decreases with *B*. The two competing effects work together in producing the non-monotonic $\Delta\rho\left( B \right)$ at 40 K.

**Figure S7 │ Dissipative superconducting vortex motion driven by the Lorentz force. a**, The longitudinal resistivity $\rho(\phi=0^{o},\gamma)$ and transverse resistivity $\rho_{T}(\phi=0^{o},\gamma)$ at *T* = 40 K and *B* = 14 T. The black open dots are experimental data and the red curves are the best fittings according to Expressions (3) and (4). **b**, The amplitude of angular oscillation, $\Delta\rho$, as a function of *B* at various *T*. $\Delta\rho$ retrieved from $\rho(\phi=0^{o},\gamma)$ (black curve) and $\rho_{T}(\phi=0^{o},\gamma)$ (red curve)

**5. Linear *I*-*V* relations above *T_c_***

The *I*-*V* curves are non-linear if the excitation current is close to *I_c_*, the critical current. This may interfere with the angle dependence of magneto-resistivity measurements. Here, we show representative *I*-*V* relations for both ***B*** // ***J*** and **B** ⊥ ***J*** measurement schemes at *T* = 38.2 K and *B* = 13 T, which corresponds to $\rho\left( \phi,\gamma=\phi\right)$ with **C_4_** symmetry in the *B*-*T* phase diagram (Fig. 4). The current used for electrical transport measurement is $I=100\mu A$, and it is apparent that the *I*-*V* curves are strictly linear from -400 $\mu A$ to 400 $\mu A$ (Fig. S8). Therefore, we can safely rule out the possibility that **C_4_** symmetry in $\rho\left( \phi,\gamma=\phi\right)$ originates from non-linear *I*-*V* dependence.

**Figure S8 │ *I*-*V* curves of LSCO film at T = 38.2 K and B = 13 T.** For both ***B*** // ***J*** and **B** ⊥ ***J*** measurement schemes ($\boldsymbol{J}$ // LSCO [100]), the I-V curves are linear for $I=100 \mu A$, which is the current used for electrical transport measurements. The solid curves are experimental data and the dashed lines are the linear fittings.

**6. In-plane versus out-of-plane anisotropy**

Superconductivity of LSCO is quasi-two-dimensional and the critical magnetic field is strongly anisotropic along the in-plane and out-of-plane directions. An example of $\rho(\theta)$ is shown in Fig. S9, where $\theta$ is the azimuth angle between ***B*** and the LSCO [100] direction. Apparently, at *T* = 35.2 K, the LSCO film is superconducting along the in-plane direction while it is resistive along the out-of-plane direction.

We took advantage of this strong anisotropy by using $\rho(\theta)$ as an indicator to calibrate the azimuth angle. By rotating the sample relative to the magnetic field while searching for the angle, at which $\rho(\theta)$ is the maximum, we can determine the angle $\theta=0^{o}$ very accurately. This guarantees the effect of the out-of-plane component of magnetic field is negligible in our measurements of in-plane magneto-resistivity.

**Figure S9 │ The magneto-resistance as a function of azimuth angle,** $\boldsymbol{\theta}$**, between the magnetic field and the LSCO [001] direction.** The experiment was conducted on a LSCO(*x* = 0.16) sample with $T_{c}^{\mathrm{off}}$ = $38.3 K$, which is slightly different from the one studied in the paper. The magnitude of the *B* field remains 7 T and its direction rotates continuously from out-of-plane to in-plane.

**7. Intrinsic C_4_ symmetry in** $\boldsymbol{\rho}\boldsymbol{(\phi,\gamma=\phi}$**)**

The **C_4_** symmetry is intrinsic to LSCO films. Here, we show more examples of $\rho(\phi,\gamma=\phi$) at fixed *B* fields and varying temperatures (Fig. S10). A transition from fourfold to twofold angular oscillations occurs as *T* increases, similar to $\rho(\phi,\gamma=\phi)$ with varying *B* fields in Fig. 2d. This clearly demonstrates that both **C_2_** and **C_4_** symmetries are intrinsic to LSCO, supporting the conclusion that nematic and *d*-wave superconductive orders are generic and intertwined in copper-based high temperature superconductors.

**Figure S10 │**$\boldsymbol{\rho(\phi,\gamma=\phi)}$ **at fixed B fields and varying temperatures.** A transition from fourfold to twofold angular oscillations occurs as *T* increases, similar to $\rho(\phi,\gamma=\phi)$ with varying *B* fields in Fig. 2d. **a**, *B* = 3 T. **b**, *B* = 7 T.

**References**

45. A. F. Hebard, A. T. Fiory, Evidence for the Kosterlitz-Thouless Transition in Thin Superconducting Aluminum Films. *Phys. Rev. Lett.* **44**, 291–294 (1980).
46. J.-P. Locquet, *et al.*, Variation of the in-plane penetration depth λ ab as a function of doping in La_2−x_Sr_x_CuO_4+δ_ thin films on SrTiO_3_: Implications for the overdoped state. *Phys. Rev. B* **54**, 7481–7488 (1996).
47. I. Božović, X. He, J. Wu, A. T. Bollinger, Dependence of the critical temperature in overdoped copper oxides on superfluid density. *Nature* **536**, 309–311 (2016).
